# Non-Invasive Measurement of Drug and 2-HG Signals Using ^19^F and ^1^H MR Spectroscopy in Brain Tumors Treated with the Mutant IDH1 Inhibitor BAY1436032

**DOI:** 10.3390/cancers12113175

**Published:** 2020-10-29

**Authors:** Katharina J. Wenger, Christian Richter, Michael C. Burger, Hans Urban, Stefan Kaulfuss, Patrick N. Harter, Sridhar Sreeramulu, Harald Schwalbe, Joachim P. Steinbach, Elke Hattingen, Oliver Bähr, Ulrich Pilatus

**Affiliations:** 1 Institute of Neuroradiology, University Hospital Frankfurt, 60528 Frankfurt am Main, Germany; elke.hattingen@kgu.de (E.H.); u.pilatus@em.uni-frankfurt.de (U.P.); 2 German Cancer Consortium (DKTK), Partner Site Frankfurt/Mainz, 60590 Frankfurt am Main, Germany; ric@nmr.uni-frankfurt.de (C.R.); michael.burger@kgu.de (M.C.B.); hans.urban@kgu.de (H.U.); patrick.harter@kgu.de (P.N.H.); sridhar@nmr.uni-frankfurt.de (S.S.); schwalbe@nmr.uni-frankfurt.de (H.S.); joachim.steinbach@kgu.de (J.P.S.); oliver.baehr@klinikum-ab-alz.de (O.B.); 3 Institute for Organic Chemistry and Chemical Biology, Center for Biomolecular Magnetic Resonance (BMRZ), Goethe University, 60438 Frankfurt am Main, Germany; 4 Department of Neurooncology, University Hospital Frankfurt, 60528 Frankfurt am Main, Germany; 5 Bayer AG, Research & Development, Pharmaceuticals, 13353 Berlin, Germany; stefan.kaulfuss@bayer.com; 6 Neuropathological Institute (Edinger-Institute), University Hospital Frankfurt, 60528 Frankfurt am Main, Germany; 7 Frankfurt Cancer Institute (FCI), 60596 Frankfurt am Main, Germany

**Keywords:** glioma, IDH mutation, 2-hydroxyglutarate, murine model, targeted therapy, small molecule inhibitor, IDH1 inhibitor, ^19^F MR spectroscopy, ^1^H MR spectroscopy

## Abstract

**Simple Summary:**

Targeted therapies are of growing interest to physicians in cancer treatment. These drugs target specific genes and proteins involved in the growth and survival of cancer cells. Brain tumor therapy is complicated by the fact that not all drugs can penetrate the blood brain barrier and reach their target. We explored the non-invasive method, Magnetic Resonance Spectroscopy, for monitoring drug penetration and its effects in live animals bearing brain tumors. We were able to show the presence of the investigated drug in mouse brains and its on-target activity.

**Abstract:**

Background: BAY1436032 is a fluorine-containing inhibitor of the R132X-mutant isocitrate dehydrogenase (mIDH1). It inhibits the mIDH1-mediated production of 2-hydroxyglutarate (2-HG) in glioma cells. We investigated brain penetration of BAY1436032 and its effects using ^1^H/^19^F-Magnetic Resonance Spectroscopy (MRS). Methods: ^19^F-Nuclear Magnetic Resonance (NMR) Spectroscopy was conducted on serum samples from patients treated with BAY1436032 (NCT02746081 trial) in order to analyze ^19^F spectroscopic signal patterns and concentration-time dynamics of protein-bound inhibitor to facilitate their identification in vivo MRS experiments. Hereafter, 30 mice were implanted with three glioma cell lines (LNT-229, LNT-229 IDH1-R132H, GL261). Mice bearing the IDH-mutated glioma cells received 5 days of treatment with BAY1436032 between baseline and follow-up ^1^H/^19^F-MRS scan. All other animals underwent a single scan after BAY1436032 administration. Mouse brains were analyzed by liquid chromatography-mass spectrometry (LC-MS/MS). Results: Evaluation of ^1^H-MRS data showed a decrease in 2-HG/total creatinine (tCr) ratios from the baseline to post-treatment scans in the mIDH1 murine model. Whole brain concentration of BAY1436032, as determined by ^19^F-MRS, was similar to total brain tissue concentration determined by Liquid Chromatography with tandem mass spectrometry (LC-MS/MS), with a signal loss due to protein binding. Intratumoral drug concentration, as determined by LC-MS/MS, was not statistically different in models with or without R132X-mutant IDH1 expression. Conclusions: Non-invasive monitoring of mIDH1 inhibition by BAY1436032 in mIDH1 gliomas is feasible.

## 1. Introduction

With 80–90% of all astrocytomas and oligodendrogliomas harboring mutations in the isocitrate dehydrogenase (IDH)1 gene, the mutant form of this enzyme is an attractive target in glioma therapy. IDH-R132X mutations lead to a gain of function, which aberrantly catalyzes the reduction of α-ketoglutarate (α-KG) to the oncometabolite, D-2-hydroxyglutarate (2-HG) [1,2,3,4]. Accumulating intracellular levels of 2-HG in cells harboring the mutated protein inhibit αKG-dependent dioxygenases, affecting the methylation status of histones as well as of cellular DNA, resulting in the glioma-CpG (5′-C-phosphate-G-3′) island phenotype (G-CIMP) and increased histone methylation marks [5]. These epigenetic changes might block the differentiation of non-transformed cells, suggesting that the mutation is a driver of tumorigenesis and can ultimately lead to unrestrained growth [3]. BAY1436032 is a selective small-molecule inhibitor of the R132X mutant form of IDH1 that selectively inhibits the mutated form of the enzyme and, thereby, reduces the aberrant generation of 2-HG [3]. Noninvasive in vivo monitoring of intracranial drug delivery is of high clinical interest, particularly in the glioma setting.

Magnetic resonance spectroscopy (MRS) offers an in vivo method for non-invasive detection of 2-HG using proton signals. This molecule has five nonexchangeable scalar-coupled protons, resonating at 4.02, 2.27, 2.22, 1.98, and 1.83 ppm, resulting in multiplets at approximately three locations. Technical difficulties include overlapping MRS-signals of 2-HG with GABA, glutamate, and glutamine, and reliable treatment response assessment requires reproducible post-processing and appropriate fitting algorithms. If these challenges are properly addressed, 2-HG can serve as a treatment biomarker [6,7,8,9,10]. Successful inhibition of the mutant enzyme using BAY1436032 should lead to a reduction in the production of 2-HG by the mIDH1 glioma cells, demonstrating the presence of the drug in the target tissue and its on-target activity.

Because BAY1436032 carries a trifluoromethoxy group, it might itself be detected using ^19^F-Nuclear Magnetic Resonance (NMR) Spectroscopy/MRS. The ^19^F nucleus resonates at a Larmor frequency that is 94% of that of ^1^H, with an NMR sensitivity of 83% relative to that of ^1^H. The endogenous ^19^F-MRS signal from the body is considered to be negligible, as physiological concentrations of detectable mobile fluorine are below the detection limit [11]. ^19^F-MRS can, therefore, provide a unique method for in vivo monitoring of drug delivery with a potentially high contrast-to-noise ratio and specificity [12,13,14,15]. However, for a detectable signal, a high density of ^19^F nuclei on the molecule and/or a high tissue concentration of the molecule are required. Compounds that are highly bound to plasma proteins have very short T2 values and are “MRS-invisible” [11]. The tissue concentration of BAY1436032 at clinical doses, the binding of BAY1436032 to plasma proteins, as well as the number of fluorine atoms present in the compound in addition to the conventional factors that determine signal to noise ratio (SNR), such as the magnetic field strength, detector design, might limit in vivo detectability.

The primary objective of this study was to show BAY1436032 target inhibition using ^1^H-MRS, monitoring changes in 2-HG/tumor total Cr (tCr) ratios in intracranial mIDH1 gliomas. The secondary objective was to evaluate the ability of in vivo ^19^F-MRS for studying drug distribution of BAY1436032, which carries a trifluoromethoxy group and is under clinical investigation in a phase I trial (NCT02746081).

## 2. Results

### 2.1. NMR Spectroscopy for Detection of BAY1436032

**Figure 1:**
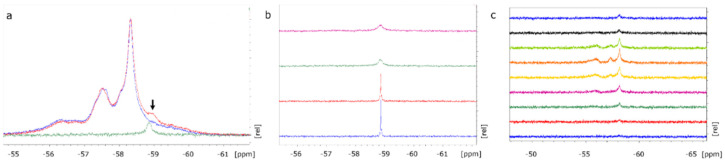
NMR spectra. (**a**) Green spectrum: HSA + BAY1436032 solubilized in DMSO and PBS; blue spectrum: plasma of patients undergoing oral treatment with IDH1 inhibitor BAY1436032 at the time of peak concentration (2 h after the last oral dose); red spectrum: same sample as green spectrum with 20 µM HSA (black arrow) added (^19^F 1D-NMR in a 3-mm tube, 210 min measurement period). (**b**) Titration of 300 µM BAY1436032 solubilized in DMSO and 600 µL PBS, with 10, 20, and 30 µM HSA (bottom to top), and no other serum proteins present, showing that the initial sharp signal peak of the unbound compound vanishes in favor of a broader albumin bound peak of BAY1436032 bound to HSA (^19^F 1D-NMR in a 3 mm tube, 30 min measurement period). (**c**) Consecutive serum samples from a patient during treatment with BAY1436032 recorded (top to bottom) pre-dose, 30 min, 1, 2, 3, 4, 6, 8, and 12 h post dose in order to demonstrate signal dynamics over time (^19^F 1D-NMR in a 3 mm tube, 7 h measurement period/probe).

Five fluorine signal peaks of BAY1436032 were recorded in patient serum samples (C_max_ at 2 h post dose) using ^19^F liquid-state 1D-NMR, with one specifically identified as albumin after titration of the serum samples with human serum albumin (HSA) (Figure 1a). Titration of BAY1436032 (in dimethyl sulfoxide (DMSO) and phosphate-buffered saline (PBS)) with HSA, and no other serum proteins present, showed that the initial sharp signal of free inhibitor shifted towards a broader signal of the HSA-bound inhibitor (Figure 1b). Serum samples from a patient treated with BAY1436032 (oral administration; Phase I Study of BAY1436032 in IDH1-mutant Advanced Solid Tumors; NCT02746081), collected consecutively over the course of 12 h during treatment showed an initial broad peak of protein-bound component. With increasing drug levels, a slow progression to the distinctive pattern of five fluorine signal peaks at C_max_ could be observed (Figure 1c). A relatively sharp peak at approximately −59 ppm included a small fraction of unbound compound. Spectra were externally referenced to trifluoroacetic acid (TFA) at −76.55 ppm (capillary probe). Signal patterns of patient serum samples were identical to those of samples with BAY1436032 (solubilized in DMSO) artificially administered to human serum probes.

### 2.2. ^19^F MRS Mouse Model

Scans of serum probes containing BAY1436032 (phantom: Eppendorf safe-lock tubes) were successfully conducted using a 7T animal scanner with sufficient SNR. In animal experiments, 3/10 mice in the GL261 group and 2/10 in the LNT-229 IDHR132H group were euthanized because of acute and severe clinical symptoms. Anatomical scans between day 23 and 33 of treatment revealed successful tumor-take in 10/10 animals for LNT-229, in 8/8 for LNT-229 IDH1-R132H, and in 7/7 for GL261. In 8/10 animals in the LNT-229 group, 7/8 in the LNT-229 IDH1-R132H group, and 4/7 in the GL261 group, adequate ^19^F spectra were obtained after adjusting field homogeneity. In animals qualifying for spectroscopic imaging, a ^19^F signal could be identified at approximately −59 to −60 ppm in the previously identified area for the BAY1436032 fluorine signal. Signal shape varied from one to multiple peaks and frequently included a relatively sharp peak and one or more broader peaks.

### 2.3. ^1^H MRS Mouse Model

**Figure 2:**
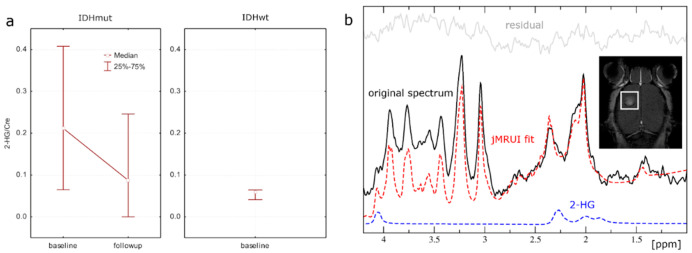
(**a**) Median and 25–75% interquartile measurements of tumor 2-HG/tCr ratios in the LNT-229 IDH1-R132H model at baseline and follow-up. (**b**) Representative murine, in vivo single voxel ^1^H-MRS spectrum for tumor tissue with IDH mutation at 7T. T2 weighted images and T1 weighted images with contrast (figure) were used for voxel placement. MRS data include original spectrum, spectral fit, residual, and the individual component 2-HG.

As a proof of the principal approach, we attempted monitoring of the pan-mutant IDH1 inhibitor BAY1436032 using ^1^H-MRS to measure tumor 2-HG/tCr ratios in the LNT-229 IDH1-R132H model. A baseline (^1^H PRESS) and a follow-up scan (^1^H PRESS and ^19^F single pulse) were performed in 7/10 mice before and after 5 days of treatment with BAY1436032 per os. After quality auditing of spectra, 5/10 mice qualified for 2-HG monitoring. Evaluation of ^1^H MRS data revealed a decrease in 2-HG/tCr ratios comparing baseline to follow-up scans (Figure 2a). Post-treatment ratios declined to ratios equivalent to those of the same cell line not transfected to express IDH1-R132H. Because of the small sample size with expected low confidence level, no statistical estimators were used. A representative ^1^H spectrum is shown in Figure 2b.

### 2.4. Mass Spectrometry

**Figure 3:**
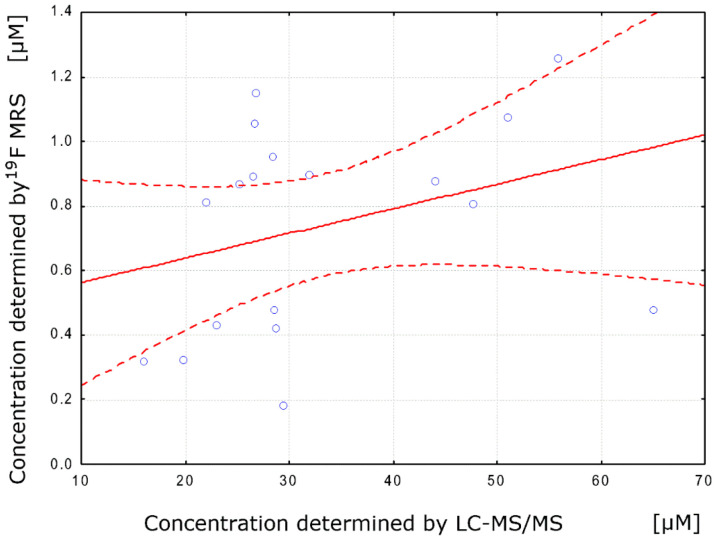
Sample Person correlation coefficient. Scatter plot (blue dots) with best-fit linear regression line (red solid line) and 95% confidence intervals (red dotted line).

Integrated ^19^F signal amplitudes (single pulse) for largely protein bound BAY1436032 inhibitor showed a low positive correlation (Pearson’s r = 0.32) with total tissue drug concentrations (tumor hemisphere and healthy tissue hemisphere) as determined by LC-MS/MS (Figure 3).

BAY1436032 concentration in the tumor tissue was not dependent on the tumor model comparing the syngeneic invasive GL261 tumor models to the slower growing LNT-229 xenograft model (*p* = 0.32; mean GL261 39.18 µM (SD 20.02), mean LNT-229 28.83 µM (SD 15.52)). Comparing models with and without the expression of the R132H mutant IDH1 for intratumoral concentrations of the mIDH1 inhibitor, we found no significant difference (*p* = 0.38; mean LNT-229 IDH1-R132H 35.63 µM (SD 13.02), mean LNT-229 28.83 µM (SD 15.52)). Mouse brains were parted in two halves (tumor hemisphere/healthy tissue hemisphere) and analyzed separately; there was no significant difference in BAY1436032 concentrations (*p* = 0.35; mean healthy tissue 29.47 µM (SD 12.49), mean tumor tissue 33.84 µM (SD 15.09)).

## 3. Discussion

In this study, we showed the presence of BAY1436032, a fluorine-containing inhibitor of the R132X-mutant isocitrate dehydrogenase and phase I study drug (NCT02746081) in the target volume of glioma mouse models and its on-target activity through direct detection of decreasing 2-HG production in mIDH1 gliomas.

Post-treatment 2-HG/tCr ratios declined to ratios equivalent to those of the same cell line not transfected to express IDH1-R132H. Because of the small sample size with expected low confidence level, no statistical estimators were used and data remains descriptive. Longitudinal treatment monitoring was applied to the LNT-229 IDH1-R132 model only, guided by the fact that merely in this model, 2-HG is produced in MRS-detectable amounts. Results are in line with prior reports in murine models and patients. Pusch et al. were able to show that 2-HG levels in subcutaneous LN229 IDH1-R132H tumors and tumors of HT1080 cells carrying a native IDH1R132C mutation could be considerably reduced after application of a single dose of 15–150 mg/kg BAY1436032 [3]. Results were obtained using a 2-HG assay after collection of tumor tissue samples. Andronesi et al. successfully used a 3D MRS imaging (MRSI) method for 2-HG detection and on target activity of the investigational drug IDH305 (Novartis Pharmaceuticals) in mIDH1 glioma patients enrolled in an open label first-in-human Phase I clinical trial (ClinicalTrials.gov identifier: NCT02381886) [16,17].

The low density of ^19^F nuclei on the molecule, preclinical evidence for a mainly protein-bound transport, and limited penetration of the blood–brain barrier (BBB) constitute specific challenges for the intracranial in vivo detection of BAY1436032. Previous experiments have shown that the maximal intraparenchymal BAY 1436032 concentration in mouse brain amounted to 38% of that in plasma levels [3]. Intracerebral tissue concentrations of lipophilic agents are predominantly controlled by plasma protein binding, active efflux, and drug metabolism [18]. Knowing that in vivo ^19^F-MRS of BAY1436032 would reveal a rather small signal, we initially identified the ^19^F protein-bound BAY1436032 signal ex vivo in serum of patients treated with the inhibitor using NMR spectroscopy. The analysis of the spectroscopic signal pattern and concentration-time dynamics facilitated in vivo identification. Protein-bound BAY1436032 showed five fluorine signal peaks. One signal could be identified as that of albumin bound BAY1436032 and there was a signal peak of free compound. Hereafter, the ^19^F-MRS signal of BAY1436032 was identified in vivo in orthotopic glioma mouse models using an optimized ^19^F single pulse sequence at a 300 MHz small animal scanner. Signal intensity was too low for acquisition of ^19^F-Images (e.g., using a fast spin echo sequence or ultra-short echo time (UTE) imaging) [19].

In addition to ^19^F single pulse MRS estimation of intracranial BAY1436032 concentration in murine models, tissue concentration was determined by LC-MS/MS. The low positive correlation of ^19^F-MRS BAY1436032 signal with actual mass spectrometric total tissue concentration (tumor hemisphere and healthy tissue hemisphere) is explained by the mainly protein-bound transport. Metabolites binding to plasma proteins either show a very fast exchange rate between bound and unbound state or a very slow exchange as for BAY1436032. The latter is reflected in short T2 relaxation times and broad line shapes for the bound part. Consequently, these “T2-shortened” signals were suppressed in our experiments [18,20,21,22,23].

Mass spectrometric tissue BAY1436032 concentrations in our model reflect total concentrations in each hemisphere, including blood and CSF compartments, after homogenization. Concentrations in blood and CSF compartments were assumed to be approximately the same, leaving tissue concentrations as the leading factor. Tissue concentrations were used to compare intracerebral BAY1436032 exposure of the syngeneic GL261 tumor model to slower growing xenograft models (LNT-229). Both models showed leakage of gadolinium-based contrast agent on T1-weighted sequences, indicating a BBB breakdown for gadolinium [24]. The LNT-229 model, derived from a primary glioblastoma (GBM), with all the hallmark mutations of primary GBM, has been shown to grow as a solid, non-infiltrative mass when implanted into the brains of immune incompetent mice. This mass excludes the normal brain parenchyma and induces neovascular growth of immature blood vessels for metabolic support. The tumor associate microvasculature lacks the protein complexes that are required to form an effective BBB. Similarly, the GL261, originally derived from a de novo mouse brain (C57BL/6NCrl background) does not exhibit diffuse single cell infiltration into the brain parenchyma, but rather it grows as a mass along abluminal surface of blood vessels and induces marked neovascularization, which again lack an effective BBB [25]. mIDH1 gliomas, on the other hand, are characterized by diffusely infiltrative, slow, indolent growth as they migrate through the brain interstitial spaces, both in grey and white matter regions. During this protracted clinical phase that can extend over 1–5 years, mIDH1 tumor cells co-opt the normal brain microvasculature for metabolic and nutritional support and do not induce microvascular proliferation or disruption of the normal BBB [26]. The drug’s ability to cross the blood–brain barrier in this protracted clinical phase can therefore not be evaluated by the employed models, which is a limitation of the study.

No significant difference in intratumoral BAY1436032 concentrations was observed between orthotopic glioma models with and without the expression of the R132H mutant form of IDH1. Taking all models into consideration, we found no significant difference in BAY1436032 concentration comparing tumor hemisphere to healthy tissue hemisphere. This very simplified approach might suggest binding of BAY1436032 to wildtype and mutant IDH1 proteins [3]. A confounding factor could be the inability to strictly separate tumor tissue from healthy tissue in the injected hemisphere.

## 4. Materials and Methods

To demonstrate target inhibition by BAY1436032, we employed ^1^H-MRS in an orthotopic glioma mouse model (LNT-229 transfected to express IDH1-R132H), and monitored changes in 2-HG/tCr ratios. The ability of in vivo ^19^F-MRS for studying drug distribution was evaluated via a three-step process:

- Ex vivo identification of ^19^F signal patterns and concentration-time dynamics of protein-bound BAY1436032 in human serum.
- In vivo identification and quantitation of BAY1436032 signal in three glioma mouse models (LNT-229, LNT-229 IDH1-R132H, GL261) following oral gavage with BAY1436032, using ^19^F-MRS.
- Ex vivo measurement of BAY1436032 by LC-MS/MS in isolated mouse brains for correlation with in vivo results.

### 4.1. NMR Spectroscopy

**Figure 4:**
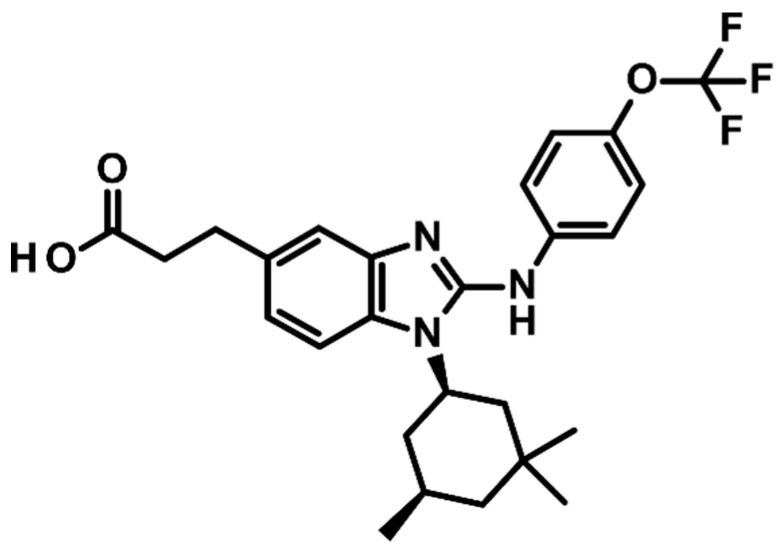
BAY1436032 with trifluoromethoxy group.

BAY1436032 (Figure 4) was solubilized in 3% DMSO or ethanol and administered to human serum, and D_2_O was added. NMR tubes contained an inner capillary tube filled with TFA (C_2_HF_3_O_2_, analytical standard) as a ^19^F reference solution (10 µL TFA in 100 µL D2O). ^19^F and ^1^H liquid-state NMR spectroscopy was conducted on these samples using Bruker NMR spectrometers operating at 500 and 600 MHz ^1^H NMR frequency (Bruker AVIIIHD, Bruker Corporation, Billerica, MA, USA equipped with TCI-Prodigy, field frequency lock: Deuterium) as well as on a small animal MR-scanner operating at 300 MHz (Bruker PharmaScan^®^, Bruker Corporation, Billerica, MA, USA). Finally, consecutive serum samples from a patient treated with BAY1436032 (oral administration; Phase I Study of BAY1436032 in IDH1-mutant Advanced Solid Tumors; NCT02746081) were collected during treatment pre-dose, and then 30 min, 1, 2, 3, 4, 6, 8, and 12 h after treatment and evaluated in the same manner.

### 4.2. ^1^H/^19^F-MRS Mouse Models

All animal experiments were performed in accordance with the National Institutes of Health Guide for the Care and Use of Laboratory Animals and institutional standards (ethical code: Gen. Nr. FK/1089). In vivo experiments were approved by the responsible government committee (Regierungspräsidium Darmstadt, Darmstadt, Germany). Twenty female athymic nude mice (Athymic Nude-Foxn1nu; Envigo) and ten female Black six mice (C57BL/6NCrl; Charles River) at the age of 5–6 weeks underwent orthotopic xenograft implantation on day 0 of the experiment using three different cell lines, LNT-229, LNT-229 transfected to express IDH1-R132H, and GL261. For each cell line, 2 μL of cell suspension (re-suspended in PBS) containing 1 × 10^5^ cells of each line were injected through a drilled opening 1 mm rostral and 2 mm lateral to the bregma in the right cerebral hemisphere. Following implantation, mice were observed twice daily and sacrificed when they developed neurological sequelae.

Anatomical scans with contrast agent (150 µL Gadovist^®^ 1.0 mmol/mL, Bayer Vital GmbH, Leverkusen, Germany) were performed on day 23 for GL261, day 26 for LNT-229, and day 33 for LNT-229 IDH1-R132H implantations. The time points were chosen according to prior experiences of our group with the respective cell lines. LNT-229 IDH1-R132H tumor bearing mice were examined using proton MRS (^1^H Point-RESolved Spectroscopy (PRESS)) on day 40. ^1^H/^19^F-MRS scans (^1^H PRESS and ^19^F single pulse) were done on day 26 for GL261, day 32 for LNT-229, and day 44 for LNT-229 IDH1-R132H. For all scans, a linearly polarized ^1^H/^19^F transmit-receive double-tuned surface coil (20 mm inner diameter), optimized for maximum SNR of ^19^F, was used. B0 shimming was applied. Acquisition parameters are listed in Table 1.

Spectra were referenced externally using a silicone capillary with 1-mm inner diameter (Carl Roth GmbH, Karlsruhe, Germany), containing TFA and distilled water (1:100). The capillary was placed centrally at the bottom of the surface coil, located between the head of the mice and the coil. All mice were anesthetized with an intraperitoneal injection of a 2% ketamine/xylazine mixture at a dose of 140 mg/kg and 10 mg/kg, respectively, to avoid the expected two peaks of isoflurane commonly used in inhalation anesthesia in the NMR spectrum [27].

**Figure 5:**
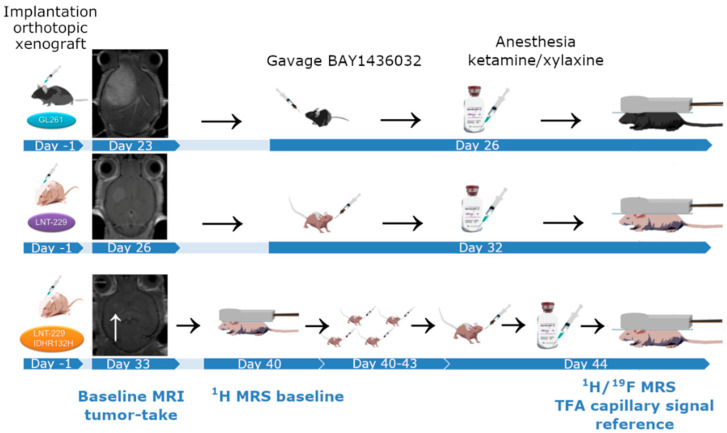
Experimental setup of the mouse models.

For animal experiments, BAY1436032 was solubilized (Vehicle: ethanol/Kolliphor HS 15^®^, Sigma Aldrich, St. Louis, MO, USA/Ampuwa, Fresenius Kabi, Bad Homburg, Germany; pH 9.2) and orally administered. Mice bearing intracranial tumors of the cell lines LNT-229 und GL261 received a single dose of BAY1436032 per os (150 mg/kg KG, Vehicle: ethanol/Kolliphor HS 15^®^, Sigma Aldrich, St. Louis, MO, USA/Ampuwa, Fresenius Kabi, Bad Homburg, Germany), 3–6 h prior to MRS, when a peak dose is suspected to occur in brain tissue according to prior experiments conducted by the distributor of the compound. Animals bearing a LNT-229 IDH1-R132H tumor model received 5 days of treatment with BAY1436032 per os (75 mg/kg KG twice per day, Vehicle: ethanol/Kolliphor HS 15^®^, Sigma Aldrich, St. Louis, MO, USA/Ampuwa, Fresenius Kabi, Bad Homburg, Germany) after baseline scan (^1^H PRESS) and underwent follow-up scan at the end of treatment (^1^H PRESS and ^19^F single pulse). Experimental set up is visualized in Figure 5. Voxel volumes of 3.0 × 3.0 × 3.0 mm^3^ were selected from the tumor area for ^1^H PRESS. Anatomical voxel position was identical for baseline and follow-up scans (visual comparison in three planes). Immediately after completion of the scan, rodents were euthanized under anesthesia using cervical dislocation. Brains were immediately recovered, parted in two halves (tumor hemisphere/healthy tissue hemisphere), and rapidly frozen in liquid nitrogen.

### 4.3. Mass Spectrometry

Brains from animals treated with BAY1436032 were homogenized with 50 mM Tris-HCl buffer, pH 7.5 (1:5 *w*/*v*) using a TissueLyser II (Qiagen^®^, Hilden, Germany). Homogenates were precipitated with acetonitrile (1:5, *v*/*v*) and frozen at −20 °C overnight. Samples were subsequently thawed and centrifuged at 3000 rpm, 4 °C for 20 min. Aliquots of the supernatants were taken for analytical measurement of BAY1436032 by liquid chromatography-mass spectrometry (LC-MS/MS). Concentrations were calculated using a corresponding calibration curve in brain homogenate and expressed in µmol/L, assuming 1 kg brain tissue equals 1 L. Results are shown in Table S1.

### 4.4. Postprocessing and Signal Quantitation

Spectra were audited by an experienced MR specialist (UP; >10 years of experience in MR spectroscopy) for quality and those of insufficient quality (large linewidth, insufficient SNR, or large artifacts at visual inspection) were not included for the analysis.

Data analysis was performed with jMRUI (JAVA-based magnetic resonance user interface, version 5.2) [28], a freely available tool, fitting in the time domain. The remaining water signal was removed using HLSVD-PRO (Hankel Singular Value Decomposition algorithm), based on the Lanczos algorithm [29]. ^19^F signals were modeled by a linear combination of Lorentzian components (AMARES = Advanced Method for Accurate, Robust, and Efficient Spectral fitting) [30], assuming a sharp signal for the free component and a larger, broad signal for the protein bound components. The latter was summarized as one signal with respect to in vivo SNR. ^1^H signals were modeled by a linear combination of simulated metabolite profiles (AQSES = Accurate Quantitation of Short Echo time domain Signals, splines configuration tight baseline constraints 9/10, points 1024) [31]. Metabolites included in murine data evaluation were 2-HG, alanine, ascorbate, aspartate, beta-glucose, creatine, glutamate, glutamine, glutathione, glycerophosphocholine-choline, glycine, lactate, myoinositol, N-acetylaspartate, N-acetylaspartylglutamate, phosphocholine, phosphocreatine, phosphoethanolamine, scylloinositol, and taurine.

### 4.5. Statistical Analysis

Pearson’s correlation coefficient (r), as a measure of the strength of the association between the two variables, was used to compare ^19^F signal amplitudes of BAY1436032 and total tissue concentration as determined by LC-MS/MS. Concentrations in tumor hemispheres, healthy tissue hemispheres, and total brain concentrations (all determined by LC-MS/MS) were compared using a two-tailed unpaired *t*-test. Results were considered to be significant at *p* < 0.05. Statistical analysis was performed using STATISTICA (version 7.1, StatSoft, Tulsa, OK, USA).

## 5. Conclusions

Noninvasive in vivo monitoring of intracranial drug delivery is of high clinical interest, particularly in the glioma setting. While technical requirements for monitoring of 2-HG in patients undergoing targeted therapy are met [10], we are currently working on a translational approach of intracranial detection of the small-molecule pan-mutant IDH1 inhibitor in patients with a clinical scanner at 3T using ^19^F-MRS.

## Figures and Tables

**Table 1:** Acquisition parameters for ^1^H/^19^F-MR-spectroscopy mouse model.

Sequence	Voxel Size	TR; Flip Angle	TE	Bandwidth Sampling Points	Scan Time	MR Frequency (MHz)
^1^H single voxel PRESS sequence	3.0 × 3.0 × 3.0 mm PRESS localized volume	2500 ms; 90°	16.5 ms	3300 Hz; 2048	2 min, 40 s	300.326
^19^F single pulse free induction decay (FID)	-	1000 ms; 60°		29.762 Hz; 4096	34 min, 8 s	282.572

## Supplementary Materials

The following are available online at https://www.mdpi.com/2072-6694/12/11/3175/s1, Table S1: BAY1436032 application in mice bearing intracranial tumors of the cell lines LNT-229 und GL261 and total tissue drug concentrations of recovered brains (tumor hemisphere and healthy tissue hemisphere) as determined by LC-MS/MS.
